# The Ecology of Care in Parkinson's Disease

**DOI:** 10.1002/mdc3.70705

**Published:** 2026-06-09

**Authors:** Joaquin A. Vizcarra

**Affiliations:** ^1^ Department of Neurology University of Pennsylvania Perelman School of Medicine Philadelphia PA USA; ^2^ Center for Neuroengineering and Therapeutics University of Pennsylvania Philadelphia PA USA

**Keywords:** access, artificial intelligence, epidemiology, health services research, Parkinson's

## A Growing Population within a Constrained System

Parkinson's disease (PD) is the fastest‐growing neurodegenerative condition worldwide. The global number of people living with PD increased from 2.5 million in 1990 to 11.8 million in 2021, and projections suggest 25.2 million by 2050.^1^ In the United States alone, approximately 1.04 million people were estimated to be living with diagnosed PD in 2017.^2^ These numbers carry direct implications for care delivery: the absolute size of the PD population is expanding within a health system of finite capacity.^1^ This growth translates into rising demand for diagnosis, longitudinal management, rehabilitation, hospital care, long‐term care, and palliative care, placing increasing pressure on the infrastructure required to deliver these services. What has been termed the Parkinson's pandemic, therefore, reflects a systems‐level challenge.^3^ Aging demographics, declining competing mortality, and expanding case ascertainment all contribute to this growth,^4^ while the workforce and care pathways responsible for meeting these needs remain constrained.

This framing reshapes the practice of movement disorders. The front door to PD care is widening as more individuals enter the diagnostic pathway, yet care remains unevenly distributed across settings, populations, and geographies. Most people with PD receive care outside the settings where innovations are developed and tested, creating a structural mismatch between discovery and delivery. This gap is reinforced by a top‐down diffusion model, in which advances from specialized centers are expected to trickle into broader community practice. Big data and artificial intelligence (AI) offer an opportunity to invert this model by integrating innovation directly into the care pathways patients actually follow.

## The Ecology of Parkinson's Disease Care Narrows Quickly and Unevenly

Green and colleagues’ revisited ecology of medical care remains one of the most useful descriptions of how populations move through the health system.^5^ Its enduring value lies in the simple observation that the settings most intensively studied in medicine are often not where most people receive care. PD provides a particularly clear illustration of this principle.

Using published US estimates, the annual ecology of PD care narrows rapidly (Fig. 1). For every 1000 people living with diagnosed PD, roughly 600 have at least one neurology visit in a year, and only 91 have at least one movement disorder specialist visit.^6^ A still smaller subset receives care through designated expert centers, and a smaller subset again participates in structured longitudinal outcomes registries.^7^, ^8^ Outside specialty care, 292 per 1000 receive PD care from primary care without neurology involvement, and 108 per 1000 are absent from identifiable primary care or neurology claims in that year.^6^

**Figure 1:**
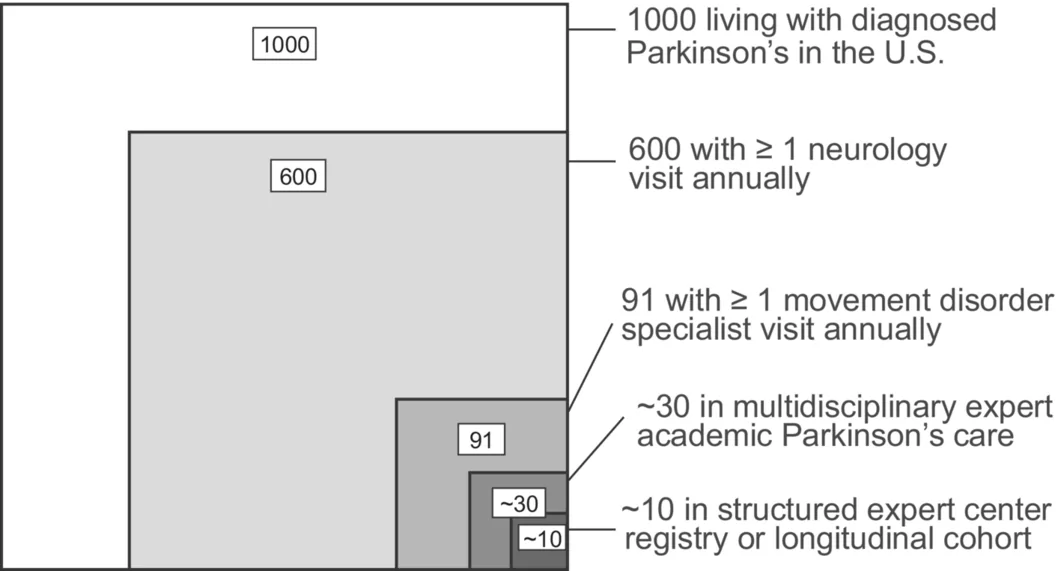
The ecology of Parkinson's disease care in the United States. Approximate annual layers per 1000 people living with diagnosed Parkinson's disease in the United States. Best‐evidence annual counts are anchored to national prevalence estimates and normalized using 2019 Medicare utilization data. Values preceded by ~ are conservative estimates rather than directly observed national utilization counts. Adapted from Green et al.

This ecology also spans care settings that extend beyond the outpatient specialty pathway. Hospitalization is common among Medicare beneficiaries with PD and varies widely by state, from 304 to 653 per 1000.^9^ Long‐term care represents a substantial component of this landscape. Nearly one quarter of a Medicare PD cohort resides in a long‐term care facility, and only one third of nursing home residents with PD receive outpatient neurologist care.^10^ Rehabilitative services remain underused, with only 203 per 1000 Medicare beneficiaries receiving physical therapy, 95 per 1000 occupational therapy, and 75 per 1000 speech‐language therapy.^6^ Family caregivers, who are predominantly spouses and often elderly themselves, bear a complex and multidimensional burden that intensifies with cognitive decline, non‐motor symptoms, and social isolation.^11^

Critically, the ecology is also unevenly distributed across populations. Black patients in the United States are nearly 30% less likely to see an outpatient neurologist, and Hispanic patients are 40% less likely, even after adjustment for demographics, insurance, and health status differences.^12^ Among Medicare beneficiaries with neurodegenerative disease, Black patients are 38% less likely to receive physical or occupational therapy.^13^ Sex‐based disparities compound these patterns: females with PD experience higher rates of depression, hip fracture, and osteoarthritis, yet use less outpatient physician care than males.^14^ Disparities in diagnosis, treatment, and research participation persist across racial and ethnic groups, limiting the generalizability of evidence and reinforcing inequitable outcomes.^15^ Taken together, these patterns suggest that the outer layers of the PD care ecology are both larger and more vulnerable. They also represent the populations least likely to be reached by current research and technological innovation.

## Earlier Detection May Widen the Front Door before Systems Are Ready

This ecological mismatch becomes more consequential as case finding moves earlier and outward. Alzheimer's disease provides a useful analogy. Blood‐based biomarkers such as plasma p‐tau217 are now being evaluated in primary and secondary care as scalable front‐door tools rather than specialist‐only tests.^16^ PD is approaching a similar trajectory. In population‐scale accelerometry analyses, wearable movement data identified PD years before clinical diagnosis.^17^ In blood‐based studies, early findings suggest that proteomic signatures may help identify individuals at increased risk before motor onset.^18^ In parallel, substantial efforts are underway to develop blood‐based alpha‐synuclein seed amplification assays.^19^

Together, these approaches point in the same direction: earlier detection, broader triage, and a growing number of individuals entering the diagnostic pathway. This shift expands demand for care and requires corresponding preparation. When case finding expands faster than care pathways, the likely result is increased demand without corresponding improvements in care delivery. The challenge extends beyond specialist availability. Many health systems lack reliable pathways for triage, follow‐up, rehabilitation, social support, longitudinal monitoring, and transitions across ambulatory, hospital, and long‐term care settings.

The ecology framework clarifies what is at stake. It places detection within a sequence of care decisions and responsibilities. After identification, patients require confirmation of diagnosis, monitoring of uncertainty, counseling in the setting of elevated risk or early symptoms, and access to appropriate longitudinal care. Many will never reach a movement disorder specialist, yet still require structured support. These are implementation challenges and scientific questions alike, because the value of earlier detection depends on the system into which detection occurs.

## Big Data and AI Should Redistribute Expertise across the Ecology

The most compelling role for big data and AI in PD lies in extending specialist‐level expertise across the full ecology of care and enabling decisions where most patients are seen. This includes identifying primary care patients who warrant expedited neurological assessment, supporting longitudinal monitoring between visits, detecting functional decline or rising hospitalization risk, triaging rehabilitative services, and enabling long‐term care clinicians to recognize patterns that would benefit from specialist input. Evidence supporting this approach already exists. A national randomized trial demonstrated that virtual specialist visits improved quality of care and reduced travel burden for people with PD who lacked local access to movement disorder expertise.^20^ What remains to be built is a layer of decision support and continuous monitoring tools capable of extending these benefits more broadly and consistently.

The economic landscape increasingly reinforces the feasibility of this approach. The Centers for Medicare & Medicaid Services Advancing Chronic Care with Effective, Scalable Solutions (CMS ACESS) Model, launching in July 2026, introduces voluntary, outcome‐aligned payments for technology‐enabled chronic disease management across several condition tracks, with annual per‐beneficiary amounts ranging from $90 to $420, depending on clinical complexity and care period.^21^ PD is not currently included, but the model's design signals the trajectory of reimbursement in the United States: scalable, technology‐enabled care coordination in which payment depends on demonstrated improvements in outcomes or biomarkers. In this context, per‐patient operational margins remain narrow, and AI‐augmented workflows may represent the most viable path to delivering care at a population scale. If similar models expand to neurodegenerative conditions, the field will require care pathways designed for outcome measurement, longitudinal engagement, and efficient specialist oversight across the full ecology of care.

This reframing also reshapes how AI should be evaluated in PD. Models developed on expertly labeled data from tertiary centers should be evaluated on both performance and implementation outcomes. Key questions include whether the development population reflects the intended deployment setting, whether validation spans community, hospital, and long‐term care environments, and whether implementation improves access, timeliness, and continuity of care in addition to discrimination performance. For system‐level applications, such as triage or monitoring, implementation outcomes may be as important as discrimination performance. Relevant measures include wait times, avoided hospitalizations, therapy utilization, caregiver burden, and equitable access across populations.

The role of movement disorder specialists within a growing care ecosystem also warrants clarification. Specialist expertise functions most effectively when distributed throughout networks rather than siloed in individual centers. Expert hubs can extend their reach through referral standards, virtual consultation, shared protocols and data, workforce training, and data‐enabled workflows.^22^ This model aligns with evidence showing that neurologist involvement is associated with lower risks of skilled nursing facility placement, hip fracture, and death.^23^ The goal is to build distributed systems of care that remain anchored in specialist knowledge while operating at a population scale.

## A Pragmatic Ecology Agenda for Parkinson's Disease

For clinicians and researchers, the ecology framework supports a pragmatic agenda. First, studies of biomarkers, AI, and digital tools should report where participants sit within the ecology of care; movement disorders center cohorts, hospital‐based cohorts, nursing home cohorts, and primary care–recruited cohorts represent distinct populations and should be analyzed accordingly. Second, implementation studies should prioritize the care layers that are largest and least supported, such as primary care without neurology involvement, hospital‐to‐home transitions, access to rehabilitation, and long‐term care. Third, expert‐center datasets should serve for creating tools and workflows that extend to settings with lower concentrations of expertise. Fourth, earlier diagnosis should be linked to improved care; expanding case finding delivers value when paired with referral pathways, counseling strategies, monitoring plans, and realistic workforce assumptions. Fifth, equity should be embedded as a design requirement; populations most affected by disparities in PD care are also those most likely to reside in the outer layers of the ecology, and tools that fail to account for this will widen existing gaps.

The ecology of care in PD defines the framework under which technological innovation succeeds or perishes. As the population grows, progress depends on whether care systems can absorb and act on new information at scale. The greatest opportunity for AI may lie in strengthening how the entire ecology operates, extending specialist expertise beyond traditional boundaries, and enabling effective care for the entire population living with PD.

## Author Roles

JAV: Conception, organization, and execution of the research project; design and execution of the statistical analysis; writing of the first draft and review and critique of the manuscript.

## Disclosures

**Ethical Compliance Statement:** This work did not involve human subjects research requiring institutional review board or ethics committee approval. Informed patient consent was not necessary for this work. I confirm that I have read the Journal's position on issues involved in ethical publication and affirm that this work is consistent with those guidelines.

## Financial Disclosures and Conflicts of Interest

Author disclosures are available in the Supporting Information.

## Supporting information


**Data S1.**COI_disclosure.

## Data Availability

Data sharing not applicable to this article as no datasets were generated or analyzed during the current study.
